# Association between Dietary Patterns and Chronic Diseases among Chinese Adults in Baoji

**DOI:** 10.1155/2014/548269

**Published:** 2014-09-21

**Authors:** Honglin Wang, Feng Deng, Meng Qu, Peirong Yang, Biao Yang

**Affiliations:** Baoji Center for Disease Control and Prevention, Xibao Road, No. 68, Baoji, Shaanxi 721006, China

## Abstract

*Objective*. This study was aimed to identify the dietary patterns among Chinese adults in Baoji and explore the association between these dietary patterns and chronic diseases. *Methods*. With multistage stratified random sampling and semiquantitative food frequency questionnaire, the prevalence of chronic disease and dietary intake was investigated in 2013. We used factor analysis to establish dietary patterns. *Results*. A total of 5020 participants over 15 years old were included in this study. Five dietary patterns were identified in Baoji named as protein, balanced, beans, prudent, and traditional patterns. There are many protective effects with protein, balanced, and beans dietary patterns on chronic diseases. *Conclusions*. We should encourage Baoji city residents to choose protein, balanced, and beans dietary patterns and abandon prudent and traditional patterns.

## 1. Introduction

According to the study of World Health Organization (WHO), cardiovascular diseases caused 33.7% of all deaths in the world, while other chronic diseases were responsible for 26.5% [1]. Globally, it has been estimated that premature deaths resulting from hypertension annually are approximately 7.1 million, which account for 64 million disability-adjusted life years (DALYs) [2]. Hypertension plays a major etiologic role in the development of ischemic heart disease, cerebrovascular disease, and cardiac and renal failure, and it is a significant risk factor for mortality and disability rates throughout the world [3].

Dietary patterns carry out extraction and analysis on dietary status overall and consider the interaction between all kinds of foods and nutrients. So dietary patterns represent a broader picture of food and nutrient consumption and may thus be more predictive of diseases risk than individual food or nutrient [4]. Furthermore, in addition to studying the role of individual nutrients, dietary patterns have become a focus for nutritional research [5]. Factor analysis of dietary patterns helps us understand the proportion of all kinds of foods in dietary intake by dimension reduction. The variable which represents correlation coefficient between foods and patterns is factor loading, and a positive loading designates positive association with the factor, while a negative loading designates negative association with the factor, and the larger the loading of a given food item to the factor, the greater the contribution of that food item to a specific factor.

Dietary habits in Asians including Chinese [6] are substantially different from those of Westerners. Therefore, it is important to examine the dietary pattern and its association with chronic diseases among people in China. Even dietary habits in Northwest China including Baoji are different from other cities. However, dietary pattern and its association with hypertension, coronary heart disease, stroke, and other chronic diseases have not been studied in Baoji city by now. Baoji, a representative city of Chinese West, the second largest city in Shaanxi province, has eight thousand years of civilization and more than two thousand seven hundred years of built history.

The present study was aimed to survey dietary and health status of the residents over 15 years old in Baoji city, identify the dietary patterns among Chinese adults in Baoji, and explore whether these dietary patterns are associated with chronic diseases (e.g., hypertension, coronary heart disease, stroke, bone and joint disease, neck and lumbar disease, and cancer). Then it could provide a scientific basis for recommendations to improve food intakes and for government to formulate nutrition policy.

## 2. Methods

### 2.1. Research Design

According to the 2010 Chinese national chronic diseases survey and the fifth National Health Service survey, we designed “The Epidemiology Questionnaire of Chronic Diseases and the Related Risk Factors in Baoji City” by many discussions, mainly including health interview, two weeks of chronic disease, the family in general, dietary, and body measurement.

### 2.2. Study Population

This study was based on population sampling survey according to the Handbook of Baoji city of Shaanxi province in China, using multistage stratified random sampling from the total Baoji population and Kish grid method, which was organized by Baoji City Health Bureau and performed by Baoji CDC. Inclusion criteria were age over 15 years old, residence in Baoji city for more than six months, participating in the study voluntarily, and actively cooperating to complete the questionnaire and measurement; exclusive criteria were age less than 15 years old, nonresidents, and not being able to match with it. A total of 5020 participants were included in this study at 12 counties, excluding the invalid samples, finally remaining 4968 valid samples. Among valid samples, men were 2519 (50.7%), women were 2449 (49.3%), and mean age (standard deviation) was 41.6 (16.3) years old, coming from the urban and rural regions, respectively, 36.7% and 63.3%. The composition of study population was in accordance with the Baoji city population of 2010 according to statistical test; therefore, we can have the generalization from this study to the general Baoji population.

### 2.3. Survey Method and Quality Control

The questionnaire and anthropometry were carried out by well-trained medical examiners. The field investigation was conducted in April 2013. Presurvey was arranged at one county before the formal investigation; it provided unified survey methods and achieved excellent effect. Height, weight, waist circumference, and blood pressure were obtained using standardized techniques and equipment in the early morning fasting state. Height was measured to the nearest 0.1 cm with the subjects standing without shoes. Weight in light clothes was measured to the nearest 0.1 kg. Body mass index (BMI) was calculated as weight divided by the square of height (kg/m^2^). Blood pressure was measured in a sitting position. Three measurements were made on all subjects at 5 min intervals, and the average of three times was used in the analysis. All subjects in the survey participated voluntarily, and written informed consent was obtained from all subjects. All aspects of design, train, sample, field investigation, data entry, and check were run through quality control.

### 2.4. Assessment of Dietary Intake

We investigated the dietary consumption frequency and quantity of participants in the past one year using semiquantitative food frequency questionnaire, converted into grams per person per day, calculated for each nutrient contained in the food with nutrition calculator software, and finally merged into daily nutrient consumption per person. Dietary questionnaire contained edible oil, spices, and 27 types of food. In the establishment of the dietary pattern, considering the statistical efficiency, similar food which was particularly small consumption got interpretation and consolidation (fresh milk, milk powder and yogurt into milk; pickles, pickled cabbage and sauerkraut into pickled vegetables; fruit juice drinks and other beverages into beverage), and finally we obtained the daily intake of 22 kinds of food.

### 2.5. Definition of Chronic Diseases

Hypertension: systolic blood pressure ≥140 mmHg or diastolic blood pressure ≥90 mmHg or previous diagnosis of hypertension, according to “Chinese Guidelines for Prevention and Treatment of Hypertension (2010 Edition)”; other chronic diseases information was collected with the self-reported questionnaire which depended on doctors above county level medical institutions diagnosis.

### 2.6. Statistical Analysis

EpiData 3.1 was used to double input data, and database was analyzed with SPSS17.0 software after check. We described measurement data by mean ± standard deviation, using *t*-test, chi-square test, and ANOVA statistical test, and all analyses performed with *P* < 0.05 were considered statistically significant. Principal component factor analysis was used to identify dietary patterns, with the factors rotated by varimax orthogonal transformation, so as to achieve simpler structure with greater interpretability [7]. Factor number was selected mainly according to the following: (1) eigenvalue > 1.0; (2) the plot pointing out distribution mainly; (3) the proportion of variance explained by each factor, but only as a reference; (4) the probability of factor extraction.

## 3. Results

### 3.1. Factor Analysis and Dietary Patterns

Kaiser-Meyer-Olkin measurement was 0.803, and Bartlett spherical test was *P* < 0.001. It is suggested that the data is adapted for factor analysis. Factor analysis extracted five dietary patterns; their eigenvalues were all larger than 1.0, and the five factors of the accumulative variance contribution rate reached 41.124% in Table 1.

Factor loadings more than 0.20 (absolute value) were selected to be analyzed, and factor was named according to the food it contained in dietary pattern to best represent the nature of each factor, respectively: protein, balanced, beans, prudent, and traditional dietary patterns in Table 2. Factor 1 was characterized as high positive loadings on fried pasta, beef and mutton, poultry, organ meats, sea foods, dried tofu, seaweed, pickled vegetables, pastry, and beverage. Factor 2 had high positive loadings on rice, fried pasta, pork, dairy, eggs, soybean milk, fresh vegetables, pastry, fresh fruit, and beverage. Factor 3 had high positive loadings on dried tofu, soybean milk, dry beans, seaweed, and fresh fruit. Factor 4 had high positive loadings on whole grains, potatoes, fried pasta, and pickled vegetables. Factor 5 showed high positive loadings on wheat, whole grains, fried pasta, pork, fresh vegetables, and pickled vegetables.

### 3.2. Demography and Body Indices by Dietary Pattern

Each factor was divided into Q1 (low percentile), Q2 (middle percentile), and Q3 (high percentile) by score; the higher the score, the more the incline to this dietary pattern. Research suggests that every kind of dietary patterns is related to demographic characteristics, different lifestyles, and movement [8]. The demography and body indices across percentiles of the dietary pattern are shown in Table 3. The average age of protein, balanced, and beans dietary patterns in Q3 was younger than in Q1, the average age of prudent pattern in Q3 was older than that in Q1, and traditional pattern was indifferent; the BMI of each pattern was indifferent. The waist circumference of protein, balanced, and beans dietary patterns in Q3 was longer than in Q1, and prudent and traditional patterns were indifferent; females of protein and beans patterns in Q3 were more than males, and males of prudent and traditional patterns in Q3 were more than females; gender of balanced pattern was indifferent.

### 3.3. Association between Dietary Pattern and Chronic Diseases Prevalence Rate

Table 4 shows seven kinds of chronic diseases according to dietary pattern.

Regarding hypertension, the prevalence rate of protein, balanced, and beans patterns in Q3 was significantly lower than in Q1, whereas the prevalence rate of prudent and traditional patterns in Q3 was higher than in Q1.

Regarding coronary heart disease, the prevalence rate of prudent pattern in Q3 was higher than in Q1, and the other four patterns were indifferent.

Regarding stroke, the prevalence rate of protein and balanced patterns in Q3 was lower than in Q1, and the other three patterns were indifferent.

Regarding bone and joint disease, the prevalence rate of balanced and beans patterns in Q3 was lower than in Q1, the prevalence rate of prudent pattern in Q3 was higher than in Q1, and the other two patterns were indifferent.

Regarding neck and lumbar disease, the prevalence rate of protein, balanced, and beans patterns in Q3 was lower than in Q1, and the other two patterns were indifferent.

Regarding cancer, the prevalence rate of protein pattern in Q3 was higher than in Q1, and the other four patterns were indifferent.

## 4. Discussion

At present, there are few researches on the association of dietary nutrition and chronic diseases of Baoji city residents, even of Shaanxi province or northwest China. Through the investigation of diet and health, we find out food nutrients intake, dietary patterns, and association of local chronic diseases. It will provide baseline data for nutritional intervention.

In this study, five dietary patterns by factor analysis were identified in a Chinese adult population named as protein, balanced, beans, prudent, and traditional patterns. The contribution rate was 16.1%, 7.9%, 7.0%, 5.2%, and 5.0%, respectively, reaching 41.1% of accumulative variance contribution rate, and it was high compared to other dietary studies of cumulative contribution rate of about 30% or less [9, 10]. In addition to factor analysis, there are other methods to identify dietary patterns (e.g., reduced rank regression (RRR)) [11]. RRR provides linear functions of predictor variables that explain as much of the variation in response variables as possible, whereas factor analysis derives dietary patterns by maximizing the explained variation of all predictor variables [12].

The demography and body indices by dietary pattern found that the age of protein, balanced, and beans dietary patterns was younger, whereas the age of prudent pattern was older; females were more in protein and beans patterns, while males were more in prudent and traditional patterns. It indicates that young people like protein, balanced, and beans dietary patterns more, and old people like prudent pattern more. There are two kinds of obesity: general obesity which takes BMI as index and abdominal obesity (also known as central obesity) which takes waist circumference as index. In this study, the BMI of each pattern was indifferent, whereas the waist circumference was longer in protein, balanced, and beans patterns. It suggests that dietary factors more easily lead to abdominal obesity than general obesity.

We also found that protein, balanced, and beans patterns were associated with reduced prevalence of hypertension and neck and lumbar disease, protein and balanced patterns were associated with reduced prevalence of stroke, balanced and beans patterns were associated with reduced prevalence of bone and joint disease, whereas prudent and traditional patterns were associated with an increased prevalence of hypertension, prudent pattern was associated with an increased prevalence of coronary heart disease and bone and joint disease, and protein pattern was associated with an increased prevalence of cancer.

A variety of studies have been implemented to explain the correlations between dietary patterns and hypertension, cardiovascular diseases, stroke, and cancer [13, 14]. Previous studies that have identified dietary patterns and their association with chronic diseases have been reported from USA [15–17], Canada [18], Germany [19], Portugal [20], Iran [21], or other Asian countries [22–24]. These studies have found that dietary patterns characterized by high intakes of vegetables, fruits, and fish were inversely associated with diseases, whereas dietary patterns characterized by high intakes of red meat, processed meat, refined grains, and fried foods were associated with increased risk.

Our results are comparable to those of previous studies. The balanced dietary pattern in present study is similar to the healthy dietary pattern identified in Western countries with a comprehensive intake of protein, carbohydrate, and fat, which are associated with the low prevalence of many chronic diseases. A systematic review study indicated that fruits and vegetables concentrates are effective in significantly improving circulating concentrations of antioxidant vitamins, provitamins, and folate and decreasing markers of oxidative stress [25], which has been associated with a reduced risk of many chronic diseases [26]. High intakes of fruits, vegetables, cereals, fishes, nuts, low-fat dairy products, and poultry in addition to relatively low intakes of fat and sugars appeared to be effective in lowering blood pressures and hypertensions [27]. A dietary pattern with frequent intakes of fruits and dairy products significantly decreased blood pressure among Chinese [28]. The Dietary Approaches to Stop Hypertension (DASH) dietary pattern, which is rich in fruit, vegetables, and low-fat dairy products and limits saturated fat, red meat, and sweets, is a success in hypertension control [29]. On the other hand, it has been reported that the increased “Western” dietary pattern is typically associated with the increased prevalence of chronic diseases such as coronary heart diseases [30] and cancer [31]. A Western dietary pattern with a high intake of red and processed meat, butter, oils, fats, sweets and desserts, refined grains, and high-fat dairy was related to a significantly increased risk of cardiovascular disease in Western populations [32]. Hu reported that the “Western diet pattern” score was positively associated with the fat intake percentage and the prevalence of coronary heart disease [33]. A cohort study in Japan also demonstrates that a dietary pattern with high meat intakes was closely related to an increased risk of cardiovascular diseases [34]. In this study, we did not identify the Western dietary pattern; however, the identified prudent pattern rich in whole grains, potatoes, fried pasta, and pickled vegetables was positively associated with the prevalence of coronary heart disease and bone and joint disease. In the present study, the protein dietary pattern was positively associated with cancer, which may be interpreted partly as high meat intakes (the first three factor loadings: organ meats 0.713, poultry 0.692, and beef and mutton 0.643). As to the traditional pattern, Baoji traditional dietary pattern rich in whole grains, fried pasta, and vegetables did not show any protective effect on chronic diseases, likely findings from a Korean study [35].

This study has several limitations. Firstly, confounding factors could not be avoided as an observational study although we have used multistage stratified random sampling. Secondly, there was some potential recall and reporting bias for semiquantitative food frequency questionnaire. Future prospective cohort and clinical trials are warranted to verify our findings.

In conclusion, five major dietary patterns were identified by factor analysis and were associated with the prevalence of many chronic diseases among Chinese adults. The protein dietary pattern was negatively associated with hypertension, stroke, and neck and lumbar disease. The balanced pattern was negatively associated with hypertension, stroke, bone and joint disease, and neck and lumbar disease. The beans pattern was negatively associated with hypertension, bone and joint disease, and neck and lumbar disease. The prudent pattern was positively associated with hypertension, coronary heart disease, and bone and joint disease. The traditional pattern was positively associated with hypertension. In the future, these results need to be confirmed by prospective cohort and clinical trials. On the whole, there are many protective effects with protein, balanced, and beans dietary patterns, so we should encourage Baoji city residents to choose protein, balanced, and beans dietary patterns and abandon prudent and traditional patterns to prevent the incidence of hypertension, coronary heart disease, stroke, and other chronic diseases.

## Figures and Tables

**Table 1:** The eigenvalue of correlation matrix.

	1	2	3	4	5
Eigenvalue	3.532	1.734	1.529	1.149	1.102
Proportion (%)	16.056	7.881	6.952	5.225	5.010
Cumulative (%)	16.056	23.937	30.889	36.114	41.124

**Table 2:** The results of dietary patterns identified by factor analysis.

Factor 1: protein		Factor 2: balanced		Factor 3: beans		Factor 4: prudent		Factor 5: traditional	
Diets	Factor loading	Diets	Factor loading	Diets	Factor loading	Diets	Factor loading	Diets	Factor loading
Fried pasta	0.204	Rice	0.490	Dried tofu	0.684	Whole grains	0.683	Wheat	0.718
Beef and mutton	0.643	Fried pasta	0.292	Soybean milk	0.480	Potatoes	0.784	Whole grains	0.292
Poultry	0.692	Pork	0.207	Dry beans	0.710	Fried pasta	0.429	Fried pasta	0.296
Organ meats	0.713	Dairy	0.572	Seaweed	0.285	Pickled vegetables	0.217	Pork	0.545
Sea foods	0.546	Eggs	0.483	Fresh fruit	0.299	—	—	Fresh vegetables	0.459
Dried tofu	0.238	Soybean milk	0.424	—	—	—	—	Pickled vegetables	0.211
Seaweed	0.228	Fresh vegetables	0.450	—	—	—	—	—	—
Pickled vegetables	0.487	Pastry	0.326	—	—	—	—	—	—
Pastry	0.294	Fresh fruit	0.623	—	—	—	—	—	—
Beverage	0.242	Beverage	0.334	—	—	—	—	—	—

—: No items.

**Table 3:** Demography and body indices by dietary pattern.

Variables	Groups	Protein	Balanced	Beans	Prudent	Traditional
Age (yr)	Q1	43.9 ± 16.2	44.1 ± 16.6	43.6 ± 16.5	40.6 ± 16.1	42.1 ± 16.8
	Q2	41.7 ± 16.7	40.6 ± 16.3	40.7 ± 16.4	41.2 ± 16.9	40.8 ± 16.2
	Q3	39.2 ± 15.7	40.1 ± 15.8	40.5 ± 15.9	43.0 ± 15.8	41.9 ± 15.8
	*F*	34.8∗	30.2∗	18.2∗	10.0∗	2.9^#^

Gender (male/female)	Q1	915/741	842/814	879/777	752/904	736/920
	Q2	791/865	867/789	842/814	911/745	814/842
	Q3	813/843	810/846	798/858	856/800	969/687
	*χ* ^2^	21.2∗	1.2^#^	7.9∗	31.5∗	68.0∗

BMI (kg/m^2^)	Q1	22.8 ± 2.9	22.9 ± 3.0	22.8 ± 2.9	22.8 ± 2.9	22.8 ± 3.0
	Q2	22.8 ± 2.9	22.8 ± 2.9	22.9 ± 3.0	22.8 ± 2.9	22.8 ± 2.9
	Q3	22.9 ± 3.0	22.8 ± 2.9	22.8 ± 2.9	22.9 ± 3.0	22.9 ± 3.0
	*F*	0.5^#^	0.7^#^	0.6^#^	0.9^#^	0.9^#^

Waist (cm)	Q1	80.0 ± 10.3	80.0 ± 9.8	80.0 ± 9.8	80.8 ± 9.8	80.7 ± 9.7
	Q2	80.5 ± 9.2	80.9 ± 10.4	80.7 ± 9.8	80.3 ± 10.1	80.6 ± 9.5
	Q3	81.1 ± 10.0	80.8 ± 9.4	80.9 ± 10.0	80.5 ± 9.7	80.3 ± 10.3
	*F*	4.6∗	4.1∗	3.6∗	1.0^#^	0.6^#^

^*^ *P* < 0.05; ^#^ *P* > 0.05.

**Table 4:** Chronic diseases prevalence rate (%) by dietary pattern.

Chronic diseases	Groups	Protein	Balanced	Beans	Prudent	Traditional
Hypertension	Q1	25.4	25.1	24.2	17.1	18.8
	Q2	19.8	20.4	19.1	22.6	19.7
	Q3	17.3	17.1	19.2	22.8	23.9
	*χ* ^2^	34.0∗	32.2∗	17.2∗	21.1∗	14.7∗

Coronary heart disease	Q1	4.1	4.5	4.2	3.3	3.1
	Q2	4.2	3.3	3.6	3.5	4.1
	Q3	3.5	4.0	4.1	5.1	4.6
	*χ* ^2^	1.3^#^	3.2^#^	1.0^#^	8.5∗	4.8^#^

Stroke	Q1	3.2	3.6	3.1	1.9	2.6
	Q2	2.5	2.2	2.5	2.3	2.7
	Q3	1.7	1.6	1.8	3.2	2.2
	*χ* ^2^	7.9∗	14.6∗	5.6^#^	5.9^#^	1.0^#^

Bone and joint disease	Q1	3.9	4.2	4.2	2.4	3.3
	Q2	3.1	3.1	2.8	3.3	2.9
	Q3	2.9	2.6	2.8	4.1	3.7
	*χ* ^2^	2.8^#^	6.7∗	7.0∗	7.5∗	1.6^#^

Neck and lumbar disease	Q1	7.3	8.0	7.7	5.4	6.8
	Q2	7.5	6.2	6.7	6.6	6.3
	Q3	4.8	5.4	5.2	7.5	6.5
	*χ* ^2^	12.5∗	9.3∗	8.5∗	5.8^#^	0.3^#^

Cancer	Q1	0.0	0.2	0.2	0.2	0.2
	Q2	0.4	0.1	0.1	0.0	0.2
	Q3	0.2	0.3	0.3	0.4	0.2
	*χ* ^2^	7.4∗	2.6^#^	1.4^#^	5.6^#^	0.2^#^

^*^ *P* < 0.05; ^#^ *P* > 0.05.
